# Exploring the Relationship between Mumps and Meteorological Factors in Shandong Province, China Based on a Two-Stage Model

**DOI:** 10.3390/ijerph181910359

**Published:** 2021-10-01

**Authors:** Yuchen Zhu, Dandan Zhang, Yuchen Hu, Chunyu Li, Yan Jia, Kaili She, Tingxuan Liu, Qing Xu, Ying Zhang, Xiujun Li

**Affiliations:** 1 Department of Biostatistics, School of Public Health, Cheeloo College of Medicine, Shandong University, Jinan 250012, China; zhuyuchenl@163.com (Y.Z.); zhangdandan_2017@163.com (D.Z.); lichunyu_biosta@163.com (C.L.); jiayan8hui@163.com (Y.J.); kailishe95@163.com (K.S.); liutx96@mail.sdu.edu.cn (T.L.); 2 Heze Center for Disease Control and Prevention, Heze 274003, China; 3 MRC Clinical Trials Unit at UCL, Institute of Clinical Trials and Methodology, 90 High Holborn, London WC1V 6LJ, UK; yuchen.hu.20@ucl.ac.uk; 4 Shandong Center for Disease Control and Prevention, Jinan 250012, China; xqepi@163.com; 5 School of Public Health, Faculty of Medicine and Health, University of Sydney, Camperdown, NSW 2006, Australia; ying.zhang@sydney.edu.au

**Keywords:** mumps, influencing factors, two-stage model, exposure–response relationship

## Abstract

Background: Small-scale studies have identified temperature and other meteorological factors as risk factors for human health. However, only a few have quantified the specific impact of meteorological factors on mumps. A quantitative examination of the exposure–response relationship between meteorological factors and mumps is needed to provide new insights for multi-city analysis. Methods: The daily recorded number of mumps cases and meteorological data in 17 cities of Shandong Province from 2009 to 2017 were collected. A two-stage model was built to explore the relationship between meteorological factors and mumps. Results: A total of 104,685 cases of mumps were recorded from 2009 to 2017. After controlling for seasonality and long-term trends, the effect of low temperature on mumps was significant at the provincial level, with a cumulative *RR* of 1.035 (95%CI: 1.002–1.069) with a 1-day lagged effect. The proportion of primary and middle school students was determined as an effect modifier, which had a significant impact on mumps (*Stat* = 8.374, *p* = 0.039). There was heterogeneity in the combined effect of temperature on mumps (*Q* = 95.447, *p* = 0.000), and its size was *I*^2^ = 49.7%. Conclusions: We have identified a non-linear relationship between mumps and temperature in Shandong Province. In particular, low temperatures could bring more cases of mumps, with certain lagged effects. More public health measures should be taken to reduce the risks when temperatures are low, especially for cities with a high proportion of primary and secondary school students.

## 1. Introduction

Mumps is an acute respiratory infectious disease caused by the mumps virus [1]. In most cases, the onset is mild and the prognosis is good, but it can also cause serious complications [2]. Mumps can be prevented by vaccines [3]. The incidence of mumps in China dropped significantly after implementing the Mumps Vaccine (MuCV) [3,4]. However, there still have been outbreaks or increased incidence of mumps [5,6,7,8,9,10]. For example, in Shandong Province, China, the incidence of mumps increased significantly in 2012 and 2013, reaching 25.33/100,000 and 24.45/100,000 respectively [11]. After 2014, the number of mumps cases declined, but the incidence is still much higher than that of other respiratory infectious diseases (such as measles) [12]. Therefore, mumps is still an urgent public health problem in Shandong Province.

Although MuCV is effective, the incidence of mumps still has obvious seasonality [1], suggesting that climate or weather changes may have an impact on the occurrence of mumps. Many studies have also shown that meteorological factors are related to the occurrence of mumps, but the effects of meteorological factors varied in different studies [1,13,14,15,16,17]. For example, Wu et al. found that both extreme high-temperature and low-temperature weather will affect the risk of mumps, but Lin et al. found that the upper and lower thresholds of average temperature are 1.2 and 24.5 °C. Only when within this range, the number of mumps cases increased with temperatures within this range [15,16]. Some studies ignored the lag time of environmental variables on the health effects, or simply including the lag time manually led to biased conclusions [16], and some studies were not able to explore the non-linear relationship between environmental variables and mumps [18]. Furthermore, different analysis methods and different study areas may have a certain impact on the results [19,20,21,22].

In recent years, the two-stage model has been widely used to explore the impact of variables on the occurrence of diseases on a larger scale [23,24,25,26], especially in the field of environmental health [19,22,27,28,29]. When the study area is large (such as a province in China), whether the exposure–response relationship between meteorological factors and mumps shows different results in different cities has not yet been clarified. Although simply averaging the data of all cities can represent the level of the entire province [13], due to the large span of different cities, the mean value may not reflect the difference in impact factors, so that it may not be possible to draw real conclusions.

This study collected meteorological data and recorded cases of mumps in Shandong Province and built a two-stage model to examine the exposure–response relationship between meteorological factors and mumps in each city. By taking geographical, demographic, and socio-economic factors as potential influencing factors, our model can be used further explore the potential source of heterogeneity of city-specific effects. Our study can provide constructive suggestions for preventing and controlling mumps in Shandong Province.

## 2. Materials and Methods

### 2.1. Study Location

**Figure 1:**
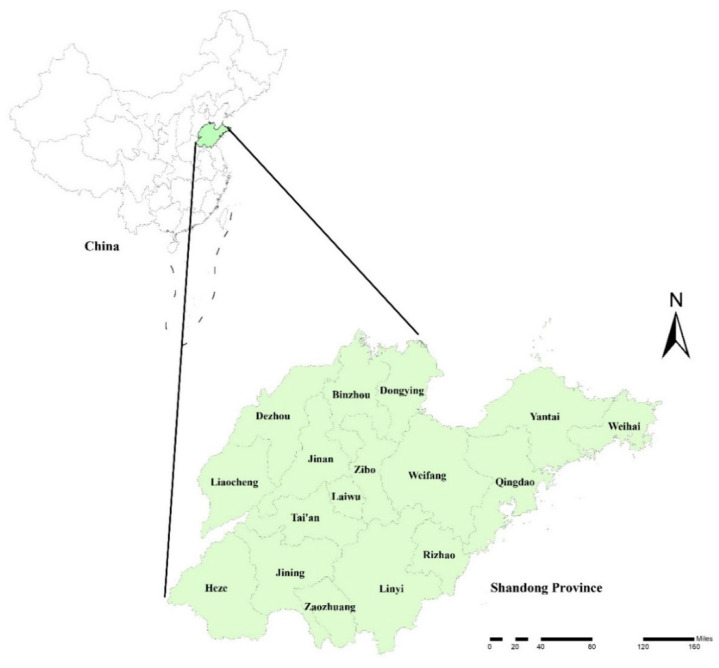
Location of the study area in China.

The study was conducted in Shandong Province, which is located on the east coast of China, between latitudes 34°25′ and 38°23′ N and longitudes 114°36′ and 122°43′ E (Figure 1). As of 2017, Laiwu City is not included in Jinan City. This study still divided Shandong Province into 17 cities.

### 2.2. Data Collection

The daily recorded data of mumps cases in 17 cities from 1 January 2009 to 31 December 2017 were collected from the Information System of the Shandong Center for Disease Control and Prevention. The daily meteorological data of 17 cities in Shandong Province were obtained from the China Meteorological Science Data Sharing Service System (http://data.cma.cn/, accessed on 27 September 2021), including the daily maximum temperature (°C) and daily average temperature (°C), daily minimum temperature (°C), daily average relative humidity (%), daily average precipitation (mm), sunshine hours (h), daily average wind speed (m/s), and daily average atmospheric pressure (hPa). The population density (person/km^2^), urbanization rate (%), GDP per capita (yuan), and the proportion of primary and middle school students (person/10,000) of each city were all derived from the Statistical Yearbook of Shandong Provincial Bureau of Statistics (http://tjj.shandong.gov.cn/tjnj, accessed on 27 September 2021).

The diagnosis of mumps is mainly based on the “Diagnostic Criteria for Mumps”, issued by the Ministry of Health of the People’s Republic of China in 2007 (http://www.nhc.gov.cn/wjw/s9491/200704/38797.shtml, accessed on 27 September2021). The epidemiological history and acute swelling of the parotid glands and (or) other salivary glands, except for parotid swelling caused by other causes, were used to make a diagnosis. Confirmed cases require laboratory-specific tests. The diagnostic criteria were consistent during the study period.

### 2.3. Two-Stage Model

In this study, the Distributed Lag Non-linear Model (DLNM) was used in the first stage to examine the non-linear relationship and the lag effects between meteorological factors and mumps by using cross-basis functions [24,27]. Meta-regression was used in the second stage to detect real exposure–response relationship at a provincial level to avoid simply averaging the data of each city.

In the first stage, quasi-Poisson was used as the connection function, and confounding factors, including long-term trend, seasonality, and day of the week, were adjusted at the same time. Spearman correlation analysis was used to test various meteorological factors to avoid multicollinearity (we excluded variables with higher correlations (*r* > 0.8) to avoid collinearity). The final model was established as follows:

(1) $$\begin{array}{r} g\left[E\left(Y_{t}\right)\right]=\alpha +\beta Temp_{t,l}+NS\left(Hum_{t,l},3\right)+NS\left(Wind_{t,l},3\right)\\ +NS\left(Sun_{t,l},3\right)+NS\left(Rain_{t,l},3\right)+NS\left(Pres_{t,l},3\right)\\ +as.factor\left(month\right)+as.factor\left(dow\right) \end{array}$$

where $Y_{t}$ represented the number of mumps cases on the *t*-th day, $Temp_{t,l}$ was the matrix obtained by applying the basis function to the daily average temperature, and β was the coefficient. NS(·) was the natural cubic spline function, and $Hum_{t,l}$, $Wind_{t,l}$, $Sun_{t,l}$, $Rain_{t,l}$ and $Pres_{t,l}$ represented the daily average relative humidity, daily average relative wind speed, daily average sunshine hours, daily average precipitation, daily average precipitation, and daily average relative atmospheric pressure, respectively.

Based on the principle of minimizing the sum of Q-AIC for every city, the degrees of freedom of each variable were 3, 3, 3, 3 and 3. month was a date variable and was included to control seasonality and long-term trends. dow was the day of the week and was used to control the day of the week. The maximum number of lag days in the model was determined by the incubation period of mumps and the principle of the minimum sum of Q-AICs in 17 cities.

In order to further explore the exposure–response relationships of multiple cities and to determine the potential sources of heterogeneity among cities, we adopted the meta-regression method in the second stage [30,31]. The heterogeneity test of meta-regression results employed Cochran’s Q test [30,32], and we used *I*^2^ to quantify the heterogeneity of the results. When *p* < 0.05 or *I*^2^ > 50%, it was considered that there was heterogeneity in each study. The independent variables were the attributes of each city included in the multivariate meta-regression, including population density, latitude, longitude, urbanization rate, GDP per capita, and proportion of students. The Wald test was used to test the influence of modifiers on the provincial effect in the meta-analysis to try to find the source of heterogeneity.

### 2.4. Model Evaluation and Sensitivity Analysis

The model residual graph was used to evaluate the fit of DLNM. We changed the degree of freedom of meteorological factors (*df* = 2–7), the maximum lag time of daily average temperature (14, 21, 30) and the degree of freedom (*df* = 6–9) of time variables, which were used to control long-term and seasonal trends for sensitivity analysis and to test the robustness of the model established by the parameters.

## 3. Results

### 3.1. Description of the Meteorological Factors, Modifiers, and Mumps Data

**Figure 2:**
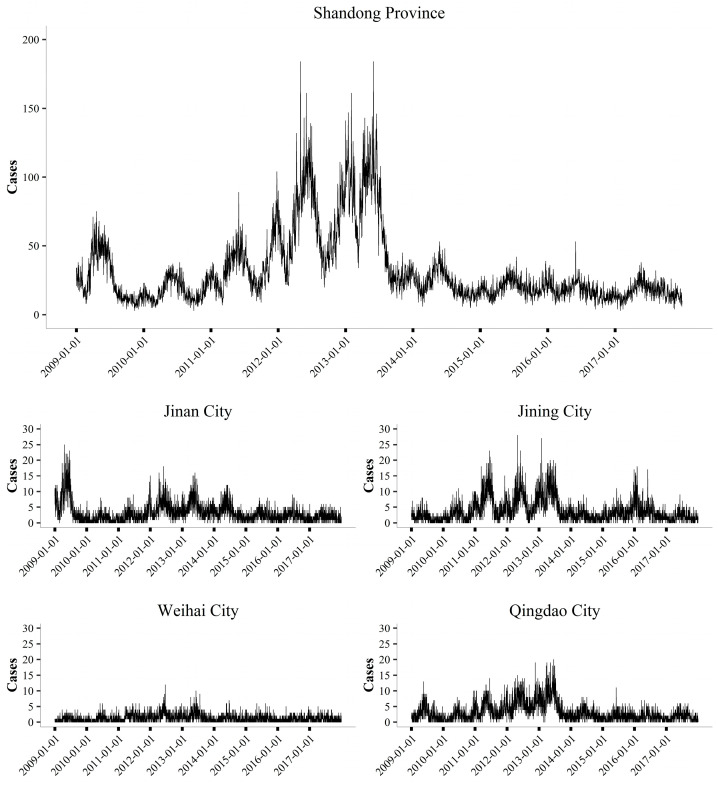
The daily cases of mumps from 2009 to 2017. The top panel represents the epidemic curve of Shandong Province, and the bottom panels represent the epidemic curves of Jinan City, Jining City, Weihai City, and Qingdao City, respectively. These four cities represent different regions of Shandong Province, China (inland and coastal cities). The epidemic curves of 17 cities are shown in the Supplementary Figure S1.

From 2009 to 2017, a total of 104,685 cases of mumps were recorded in 17 cities in Shandong Province. The overall number of cases slightly dropped before the peak values were reached in 2012/13, and then declined again (Figure 2). The temporal distribution of the cases varied greatly in different cities, but all showed a clear seasonality, with two peak periods each year (the larger peak period was from April to July, and the smaller one was from December to January).

The daily average temperatures in Shandong Province and the cities in the study period are shown in Table 1. The daily average temperatures of Shandong Province ranged from −22.80 °C to 35.00 °C, with an average of 13.37 °C. The geographic, demographic, and socioeconomic information in Shandong Province and the cities is shown in Table S1. The proportion of primary and middle school students in Heze City, Linyi City, Liaocheng City, and Zaozhuang City was higher than that at the provincial level.

### 3.2. Correlation Analysis between Meteorological Factors and Other Modifiers

The Spearman rank correlation analysis between meteorological factors and other modifiers in Shandong Province and the number of mumps cases from 2009 to 2017 is shown in Table S2. The daily cases of mumps were correlated with all meteorological factors (*p* < 0.05). According to Table S2, daily maximum temperature and daily minimum temperature were excluded to avoid collinearity due to the higher correlation coefficient (*r* > 0.80). In particular, the urbanization rate and per capita GDP were separately included in the meta-regression. Therefore, despite their high correlation (*r* > 0.8), they were not excluded.

### 3.3. The Effect of Daily Mean Temperature on Mumps

**Figure 3:**
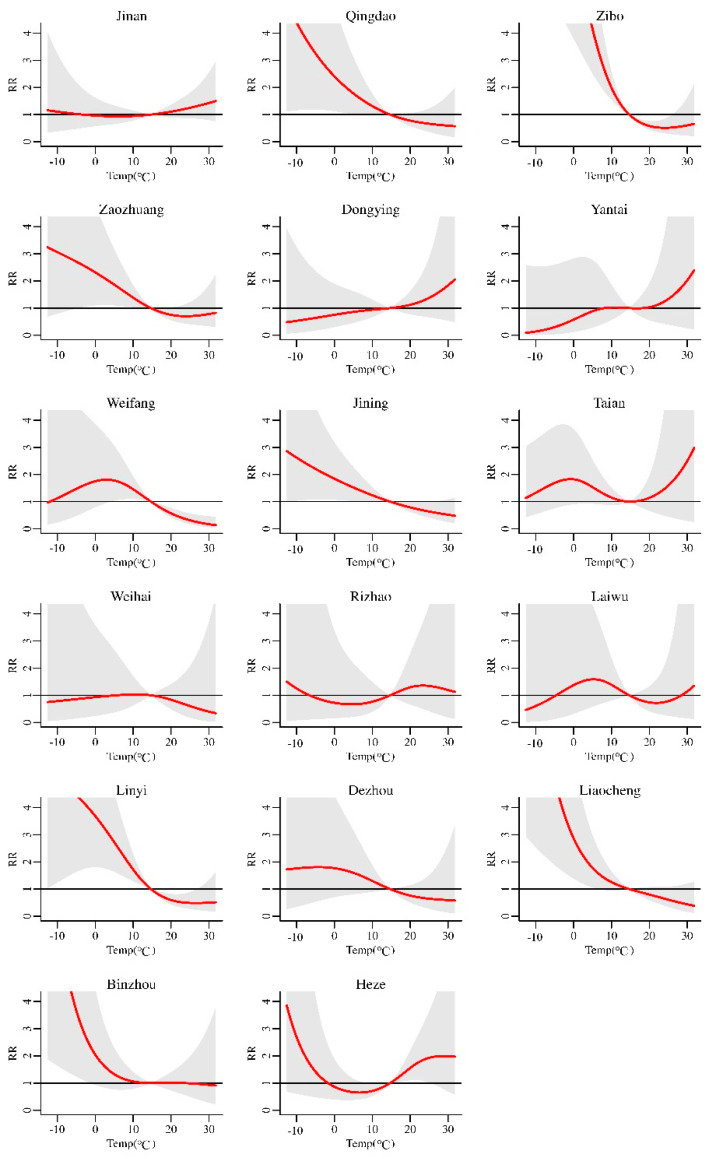
The cumulative *RR* of temperature on mumps with a 30-day lag in 17 cities in Shandong Province from 2009 to 2017.

**Figure 4:**
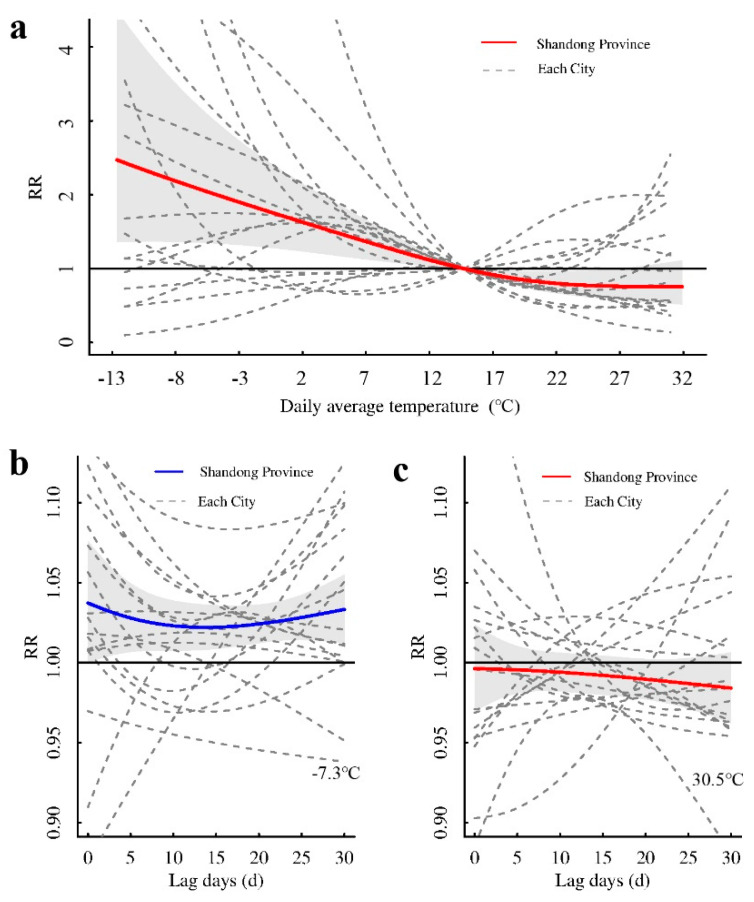
The effect of temperature on mumps. (**a**) The cumulative *RR* of temperature on mumps in Shandong Province and other cities with a 30-day lag. (**b,c**) represent the effects of low and high temperatures, respectively, in Shandong Province and 17 cities on mumps under different lag times.

In the first stage, we obtained the exposure–response relationship between the daily average temperature and the cumulative relative risk (*RR*) of mumps in 17 cities in Shandong Province, with different lag days. The exposure–response relationship when the lag time was 30 days is shown in Figure 3. It can be seen that *RR* changed with temperature (we take 14.7 °C as the reference temperature), and the exposure–response relationship varied from city to city. Most of the curves were “V”-, “L”-, or “S”-shaped. The cumulative *RR* was highest when the temperature was −12.6 °C, with an *RR* of 2.429 (95%CI: 1.339–4.407). As the daily average temperature gradually increased, the cumulative *RR* of mumps decreased. When the temperature was 29.7 °C, the cumulative RR was reduced to the lowest (*RR* = 0.759, 95%CI: 0.545–1.056), which was not statistically significant (Figure 4a).

We used *P*_1_ (1st percentile: −7.3 °C) and *P*_99_ (99th percentile: 30.5 °C) to calculate the *RR*, representing the effects of low and high temperatures on mumps, respectively. The effects of temperature on mumps at different lag times were all risk effects when the temperature was low, and there was a certain delay in the risk of onset. It appeared that when the lag time was one day, the risk was the highest (*RR* = 1.035, 95%CI: 1.002–1.069). With an increase in the lag time, the effect gradually decreased. When the lag time was 14 days, the effect value reached the minimum (*RR* = 1.022, 95%CI: 1.008–1.036), and then gradually rose (Figure 4b). The overall effect of temperature on mumps with different lag times at high temperatures was a protective effect. The longer the lag time was, the greater the protection effect. However, under different lag values (<30 days), the protective effect of high temperature on mumps was not statistically significant (Figure 4c).

In the second stage, modifying factors such as longitude and population density (Table S1) were included in the meta-regression analysis of the heterogeneity to explore the source and size of heterogeneity among cities. The heterogeneity test of the meta-regression results showed that there was heterogeneity (*Q* = 95.447, *p* = 0.000), and the size of heterogeneity was *I*^2^ = 49.7%. The results of the Wald test showed that the source of heterogeneity was the proportion of primary and secondary school students (*Stat* = 8.374, *p* = 0.039). After adding this variable in the meta-analysis, the heterogeneity dropped to 45.4%. The Wald test for the other five variables was not statistically significant (Table 2).

**Figure 5:**
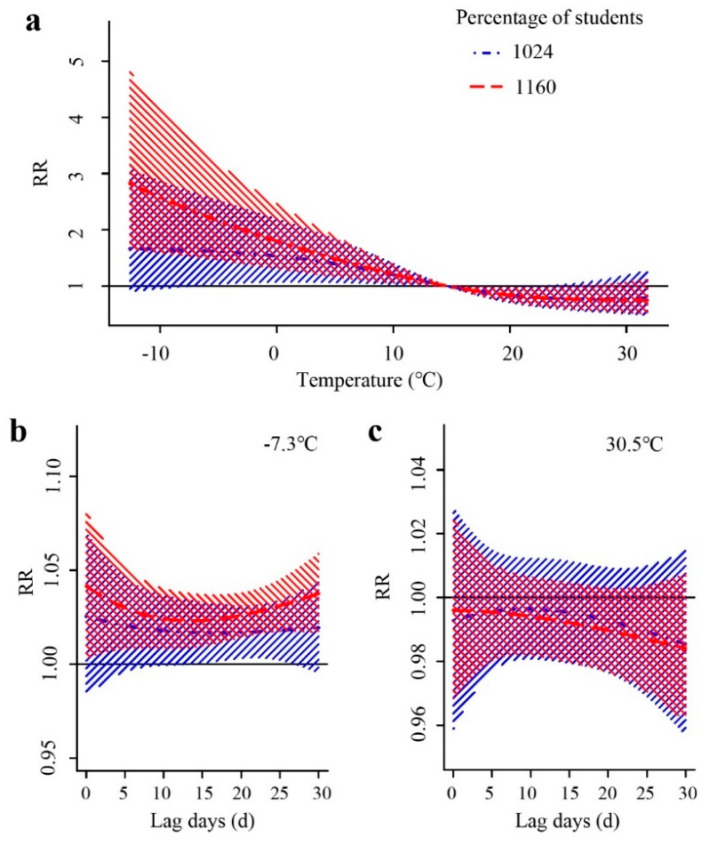
The effect of temperature on mumps when the proportion of primary and secondary school students is high (*P*_75_(1160/10,000), red) and when the proportion of primary and secondary school students is low (*P*_25_(1024/10,000), blue). (**a**) represents the effect of daily average temperature on mumps. (**b**,**c**) represent the effects of temperature on mumps at different lag times.

Based on the meta-regression model, we used *P*_25_ (1024/10,000) and *P*_75_ (1160/10,000) to represent the low and high proportions of primary and secondary school students, respectively. The exposure–response relationship of *RR* to temperature was different when the proportion of primary and secondary school students was different (Figure 5a). Compared with the median temperature of 14.7 °C, as the temperature increased, areas with a high proportion of primary and middle school students had a higher risk of mumps than areas with a low proportion. Areas with a high proportion of students had a greater risk at low temperatures, reaching a maximum value at −12.6 °C (*RR* = 2.778, 95%CI: 1.631–4.730), and then gradually decreased. The areas with a low proportion of students also had the highest risk (*RR* = 1.633, 95%CI: 0.88–3.004) at −12.6 °C, but this was not statistically significant.

When the temperature was low, cities with a high proportion of students had a greater risk than cities with a low proportion of students. There was no hysteresis in the effect of temperature on mumps, and there was no significant difference (*Stat* = 5.471, *p* = 0.140, Figure 5b). At high temperatures, the effect of the proportion of primary and middle school students at different lag times was not statistically significant (*Stat* = 0.441, *p* = 0.932, Figure 5c).

### 3.4. Model Residuals and Sensitivity Analysis

The residual distribution of the developed model had stationary random white noise, indicating that the fitting result was good (Figure S2). The results of the sensitivity analysis showed that the model established by the parameters in this study was robust and could produce credible results (Table S3). It can be seen that when we changed the degree of freedom of meteorological factors (*df* = 2–7) and the maximum lag time of daily average temperature (14, 21, 30), and the degrees of freedom of time variables were used to control long-term trends and seasonal trends (*df* = 6–9), the estimated values of the effects were all stable.

## 4. Discussion

This study found that at the provincial level, when the daily average temperature is low, the cumulative risk of mumps is higher, and the cumulative risk is low when the daily average temperature is high. The exposure–response curve of temperature to mumps differs from city to city, but most cities are similar to the provincial level. The combined effect of daily average temperature on mumps is heterogeneous. When the temperature is low, cities with a high proportion of primary and middle school students have a higher risk of mumps.

The two-stage model effectively solves the problem of loss of information by simply averaging the data, so the combined effect of daily average temperature on mumps is more accurate. However, the second stage of the two-stage model is based on the effects obtained in the first stage, so it is necessary to ensure the accuracy of the first-stage parameter estimation. In addition, the effect modifiers included in the second stage will play an important role in the results, and ignoring important effect modifiers will also bias the results. We will further explore the above issues in a future study.

The impact of temperature on mumps may vary depending on the study areas and analysis methods [19,20,21]. Onozuka et al., Ho et al., and Zhang et al. all found that temperature can affect the incidence of mumps [1,14,33]. Onozuka et al. concluded that there is a positive linear correlation between temperature and mumps. Both Ho et al. and Zhang et al. found a non-linear relationship between temperature and mumps, but the non-linear relationship that they obtained was not consistent. Ho et al. used Poisson regression to research Taiwan Province, China, but they did not consider the impact of lag time. Zhang et al. used Boosting Regression Tree (BRT) to research Jining City, China, but the dependent variable of the study was the months with high incidence of mumps rather than the number of cases. Different study areas and analysis methods may be the reasons behind the different conclusions.

This study has found that the exposure–response relationship between the daily average temperature and the cumulative relative risk of mumps at the provincial level is approximately “L”-shaped. This means that when the temperature is low, the risk of mumps is high. The biological mechanism of the effect of low temperature on mumps is unclear. However, the study of Eccles et al. has shown that cooling air increases the viscosity of the mucus layer covering the surface of the nasal mucosa and reduces the frequency of cilia swinging, which is not conducive to the excretion of adsorbed dust, harmful bacteria, viruses, etc., and easily causes nasal mucosal infection. Another possibility is that the cooling of the mucosal layer makes the virus residing in the upper respiratory tract more stable and increases the chance of infection [34]. Moreover, individuals are less likely to go outside when the temperature is low. A confined environment may increase the possibility of virus transmission. At a high temperature, the decline in incidence may be due to the inability of the mumps virus to withstand high temperatures [2], which affects the transcription and replication of the virus. Therefore, relevant government departments should strengthen the prevention and control of mumps when the temperature is low.

By adding potential influencing factors to our analysis, we found that when the temperature is low, the impact of daily average temperature on mumps is significantly higher in cities with a higher proportion of primary and middle school students than in cities with a lower proportion. Primary and middle school students are often situated in densely populated places such as classrooms with poor air circulation, which is conducive to the spread of the virus. Cities with a higher proportion of primary and middle school students mean more susceptible people. Exposure and contact between susceptible people increase the risk of mumps. On the other hand, with the improvement of the social economy and people’s living standards, the physique of Chinese children and adolescents has improved significantly, but the physical development has been relatively slow or even decreased [35]. Therefore, it is necessary to implement early warnings and early interventions in areas with a high proportion of primary and secondary school students during the epidemic season of mumps. Strengthening school health education and enhancing the physical fitness of primary and secondary school students can effectively reduce clusters of cases or outbreaks.

It is worth noting that the proportion of primary and middle school students can only explain part of the heterogeneity of cities, and limitations exist in the analyses. The unexplainable heterogeneity may be attributed to the following reasons. First, the city-specific features are not enough to fully represent their modification effect. For example, the GDP per capita was analyzed using only annual data, which provided limited information for the time series analysis. Second, there may be other important effect modifiers that we did not consider. For example, local lifestyles, public health interventions, and differences in data quality are also important factors that explain some parts of the heterogeneity [36]. Third, we used the number of daily recorded cases of mumps, which would increase the noise of the data. Although we used a variety of methods to control this in the model, it may still have had a certain impact. In addition, if we can obtain more information about the sociodemographic structure of the population, we can obtain a clearer result by excluding cities with large differences in the sociodemographic structure of the population.

One more factor that may influence the number of mumps cases is the MuCV vaccination rate of children. During the study period, Shandong Province actively implemented child immunization procedures in the National Immunization Program [37]. All the children were required to be vaccinated, except for those who could not be vaccinated due to special reasons. According to the report of the Shandong Center for Disease Control and Prevention, the vaccination rate of MuCV in children with one dose of Shandong Province was above 95% in almost all the years [38]. Therefore, considering that the children’s MuCV vaccination rate was less variable throughout the study period, it was excluded when fitting the models in the current study.

In the context of climate change, the frequency of extreme weather is increasing, and meteorological factors such as temperature will increase the risk of mumps. Future work should pay more attention to mumps when the temperature is low, especially cities with a high proportion of primary and secondary school students. Specifically, first, while routine immunization has been implemented, it is necessary to pay attention to leak detection. The enrollment vaccination certificate of all the students should be inspected when the new semester begins. Second, the mumps antibody-positive rate of citizens, especially children aged 1–15, could be spot-checked regularly. Once a low mumps antibody-positive rate is detected in some specific populations, replanting work should be implemented [38]. Third, in the cold season, namely in the autumn and winter, teachers should organize outdoor activities for students. When the students are in a closed environment (such as classrooms), the windows should be opened regularly to enhance air circulation. Finally, knowledge popularization activities could be carried out for elementary and middle school students to increase their awareness about protective measures such as maintaining social distancing, strengthening their physical fitness, etc.

## 5. Conclusions

In Shandong Province, China, there is a non-linear relationship between the cumulative relative risk of mumps and daily average temperature, with different lagged effects. At the provincial level, low temperatures could bring more cases of mumps with certain lagged effects. More public health measures should be taken to reduce the risks when temperatures are low, especially for cities with a high proportion of primary and secondary school students.

## Figures and Tables

**Table 1:** Daily average temperatures in Shandong Province and 17 cities from 2009 to 2017.

City/Province	Daily Average Temperature (°C)					
	$\bar{x}$	Min	P25	M	P75	Max
Jinan	14.92	−12.40	5.40	16.60	24.10	35.00
Qingdao	13.36	−11.50	4.80	14.50	21.80	31.20
Zibo	13.22	−13.10	3.60	14.90	22.80	32.00
Zaozhuang	14.49	−11.70	5.10	16.00	23.70	32.80
Dongying	14.03	−13.20	3.70	15.80	24.10	34.30
Yantai	12.87	−11.60	3.55	14.10	22.00	30.90
Weifang	13.72	−13.00	3.70	15.30	23.50	33.40
Jining	14.22	−10.27	4.70	15.70	23.60	32.70
Taian	6.30	−22.80	−2.30	8.00	15.10	23.00
Weihai	11.90	−10.40	3.65	12.70	20.30	27.70
Rizhao	13.87	−11.40	5.10	15.00	22.35	33.10
Laiwu	14.37	−13.10	4.60	16.20	24.00	33.70
Linyi	14.35	−11.00	5.30	15.70	23.20	32.50
Dezhou	13.44	−14.30	3.30	15.10	23.20	32.60
Liaocheng	14.21	−10.90	4.60	15.70	23.60	32.90
Binzhou	13.49	−15.30	3.30	15.20	23.40	32.90
Heze	14.46	−9.20	5.30	15.90	23.40	32.00
Shandong	13.37	−22.80	4.00	14.70	22.60	35.00

Note: Min, P25, M, P75, and Max represent the minimum, 25th percentile, median, 75th percentile, and maximum value, respectively.

**Table 2:** The heterogeneity test and Wald test results of the meta-analysis for the effect of daily average temperature on mumps.

	Cochran Q Test			*I* ^2^	Information Criterion		Wald Test		
	*Q*	*df*	*p*	*(*%*)*	*AIC*	*BIC*	*Stat*	*df*	*p*
Intercept	95.447	48	0.000	49.7	177.271	194.112	-	-	-
Longitude	88.007	45	0.000	48.9	183.815	205.495	4.026	3	0.259
latitude	89.833	45	0.000	49.9	185.137	206.812	2.826	3	0.419
Population density	94.459	45	0.000	52.4	214.426	236.106	1.803	3	0.614
Urbanization rate	90.732	45	0.000	50.4	193.855	215.535	3.684	3	0.298
GDP per capita	91.648	45	0.000	50.9	245.327	267.007	3.149	3	0.369
Proportion of students *	82.442	45	0.001	45.4	209.639	231.319	8.374	3	0.039

Note: * The proportion of students represents the proportion of primary and secondary school students in the entire population.

## Data Availability

The daily recorded data of mumps cases in 17 cities from 1 January 2009 to 31 December 2017 were collected from the Information System of the Shandong Center for Disease Control and Prevention. The daily meteorological data of 17 cities in Shandong Province were obtained from the China Meteorological Science Data Sharing Service System (http://data.cma.cn/, accessed on 27 September 2021). The socioeconomic factors of each city were all from the Statistical Yearbook of Shandong Provincial Bureau of Statistics (http://tjj.shandong.gov.cn/tjnj, accessed on 27 September 2021).

## Supplementary Materials

The following are available online at https://www.mdpi.com/article/10.3390/ijerph181910359/s1, Table S1: Geography, population, and socioeconomic variables in Shandong Province and 17 cities. Table S2: Spearman rank correlation analysis of meteorological, demographic, and other factors and the incidence of mumps in Shandong Province from 2009 to 2017. Table S3: Sensitivity analysis (relative risk RR). Figure S1: The daily cases of mumps from 2009 to 2017 in 17 cities, Shandong Province. Figure S2: The residual graph of DLNM.
