# Supplementary material for: Exploring the Relationship between Mumps and Meteorological Factors in Shandong Province, China Based on a Two-Stage Model
Source: Int J Environ Res Public Health. 2021 Oct 1;18(19):10359. doi: 10.3390/ijerph181910359 (PMC8508524; doi:10.3390/ijerph181910359)
Supplement: Supplementary file 1 [file ijerph-18-10359-s001.zip › ijerph-1393599-supplementary.pdf]

Tables

Table S1 Geography, population, and socioeconomic variables in Shandong Province and 17 cities

City	Longitude	latitude	Population density (person/km2)	Proportion of students* (/10000)	Urbanization rate	GDP per capita
Jinan	117.13	36.66	915	1160	70.53	98967
Qingdao	120.39	36.07	823	1093	72.57	119357
Zibo	118.06	36.82	789	1082	70.26	101781
Zaozhuang	117.33	34.82	859	1333	57.32	59110
Dongyong	118.59	37.45	261	783	67.75	177376
Yantai	121.45	37.47	512	844	63.66	103706
Weifang	119.17	36.71	580	1091	59.95	62592
Jining	116.59	35.42	749	1130	57.12	55595
Tai'an	117.09	36.21	727	973	60.63	63555
Weihai	117.23	31.83	487	887	66.46	123301
Rizhao	119.53	35.42	544	1062	58.65	68848
Laiwu	117.67	36.21	613	1024	62.58	65122
Linyi	118.36	35.11	614	1345	57.4	41372
Dezhou	116.37	37.44	560	1110	55.57	54197
Liaocheng	115.99	36.46	703	1344	50.34	50641
Binzhou	117.23	31.83	427	1032	58.63	66970
Heze	115.49	35.24	719	1479	49.05	32493
Shandong Province			634	1157	60.58	72851

*: The proportion of students is the proportion of primary and secondary school students.

Table S2 Spearman rank correlation analysis of meteorological, demographic and other factors and the incidence of mumps in Shandong Province from 2009 to 2017

	Precip	Pres	Wind	Hum	Sun	Mean Temp	Min Temp	Max Temp	Lon	Lat	Pop	Student	Urban	GDP	Cases
Daily precipitation (mm)	1.00														
Daily average atmospheric pressure (hPa)	-0.19*	1.00													
Daily average wind speed (m/s)	0.04*	-0.11*	1.00												
Daily average relative humidity (%)	0.47*	-0.16*	-0.16*	1.00											
Daily average sunshine hours (h)	-0.43*	-0.10*	0.10*	-0.48*	1.00										
Daily average temperature (℃)	0.15*	-0.54*	-0.13*	0.28*	0.18*	1.00									
Daily average minimum temperature (℃)	0.23*	-0.55*	-0.09*	0.38*	0.06*	0.98*	1.00								
Daily average maximum temperature (℃)	0.09*	-0.52*	-0.18*	0.20*	0.26*	0.98*	0.93*	1.00							
Longitude	0.00	0.09*	0.03*	-0.02*	-0.01*	0.01^{a}	0.04*	-0.02*	1.00						
Latitude	-0.01*	-0.07^{a}	-0.06*	-0.13*	0.04*	0.00	-0.02*	0.02*	0.22*	1.00					
Population density (Person/ square kilometer)	0.00	-0.43*	-0.10*	-0.04*	-0.03*	0.01	0.00	0.02*	-0.24*	-0.07*	1.00				
Proportion of primary and secondary school students (/10000)	0.02*	0.03*	-0.29*	0.04*	-0.06*	0.08*	0.05*	0.12*	-0.46*	-0.29*	0.61*	1.00			
Urbanization rate (%)	0.01a	-0.24*	0.27*	-0.09*	0.01	-0.03*	0.01^{a}	-0.08*	0.60*	0.31*	0.05*	-0.57*	1.00		
GDP per capita (Yuan)	0.00	-0.07*	0.26*	-0.04*	-0.03*	-0.03*	0.02*	-0.08*	0.59*	0.24*	-0.27*	-0.77*	0.88*	1.00	
Number of cases	0.02*	-0.14*	0.01^{a}	0.03*	0.05*	0.07*	0.07*	0.08*	-0.13*	-0.09*	0.29*	0.22*	0.01^{a}	-0.07*	1

Note: *: P<0.01; ^{a}: P<0.05

Table S3 Sensitivity analysis (relative risk RR)

	Daily average temperature (°C)				
	-12.6	-1.5	9.7	20.8	31.9
Daily average temperature					
df=2	2.379	1.612	1.138	0.879	0.731
**df=3**	**2.429**	**1.804**	**1.227**	**0.820**	**0.760**
df=4	2.225	1.840	1.239	0.841	0.752
df=5	1.879	1.791	1.181	0.823	0.778
df=6	2.425	1.701	1.302	1.035	1.078
df=7	3.075	1.671	1.262	0.981	0.981
Daily average relative humidity					
df=2	2.441	1.806	1.226	0.821	0.757
**df=3**	**2.429**	**1.804**	**1.227**	**0.820**	**0.760**
df=4	2.439	1.808	1.228	0.821	0.763
df=5	2.450	1.812	1.228	0.820	0.761
df=6	2.444	1.813	1.229	0.819	0.760
df=7	2.441	1.809	1.228	0.820	0.761
Daily precipitation					
df=2	2.429	1.804	1.227	0.820	0.760
**df=3**	**2.429**	**1.804**	**1.227**	**0.820**	**0.760**
df=4	2.429	1.804	1.227	0.820	0.760
df=5	2.409	1.088	1.227	0.820	0.760
df=6	2.357	1.778	1.227	0.820	0.760
df=7	2.402	1.793	1.227	0.820	0.760
Daily average atmospheric pressure					
df=2	2.473	1.838	1.238	0.813	0.753
**df=3**	**2.429**	**1.804**	**1.227**	**0.820**	**0.760**
df=4	2.440	1.805	1.226	0.823	0.768
df=5	2.432	1.801	1.225	0.822	0.764
df=6	2.399	1.792	1.225	0.822	0.767
df=7	2.392	1.783	1.221	0.826	0.773
Daily average sunshine hours					
df=2	2.423	1.803	1.227	0.820	0.760
**df=3**	**2.429**	**1.804**	**1.227**	**0.820**	**0.760**
df=4	2.455	1.814	1.229	0.820	0.760
df=5	2.429	1.805	1.227	0.821	0.762

df=6	2.459	1.819	1.230	0.818	0.759
df=7	2.452	1.822	1.233	0.816	0.752
Daily average wind speed					
df=2	2.417	1.802	1.227	0.820	0.758
**df=3**	**2.429**	**1.804**	**1.227**	**0.820**	**0.760**
df=4	2.420	1.799	1.226	0.821	0.760
df=5	2.420	1.796	1.224	0.822	0.760
df=6	2.432	1.798	1.225	0.822	0.759
df=7	2.418	1.791	1.222	0.824	0.765
Time variable					
df=6	1.288	1.398	1.196	0.827	0.817
**df=7**	**2.429**	**1.804**	**1.227**	**0.820**	**0.760**
df=8	1.987	1.474	1.118	0.874	0.670
df=9	1.348	1.273	1.106	0.889	0.789
Maximum lag time					
df=14	1.183	1.117	1.077	0.944	1.049
df=21	1.123	1.195	1.195	0.831	0.870
**df=30**	**2.429**	**1.227**	**1.227**	**0.820**	**0.760**

Figures

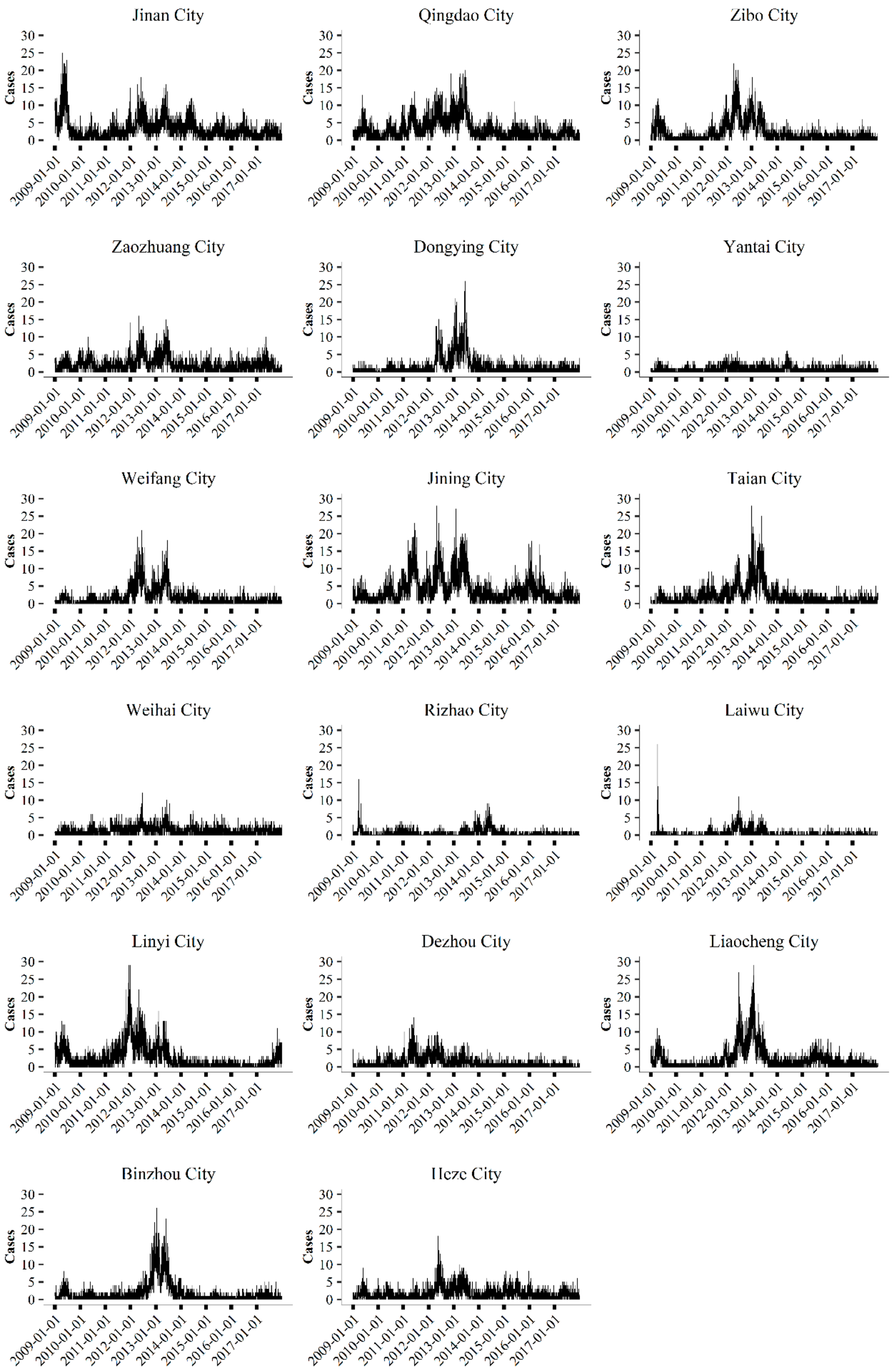
Jinan City
Qingdao City
Zibo City
Zaozhuang City
Dongying City
Yantai City
Weifang City
Jining City
Taian City
Weihai City
Rizhao City
Laiwu City
Linyi City
Dezhou City
Liaocheng City
Binzhou City
Heze City
Cases
0
5
10
15
20
25
30
2009-01-01
2010-01-01
2011-01-01
2012-01-01
2013-01-01
2014-01-01
2015-01-01
2016-01-01
2017-01-01

Figure S1. The daily cases of mumps from 2009 to 2017 in 17 cities, Shandong province.

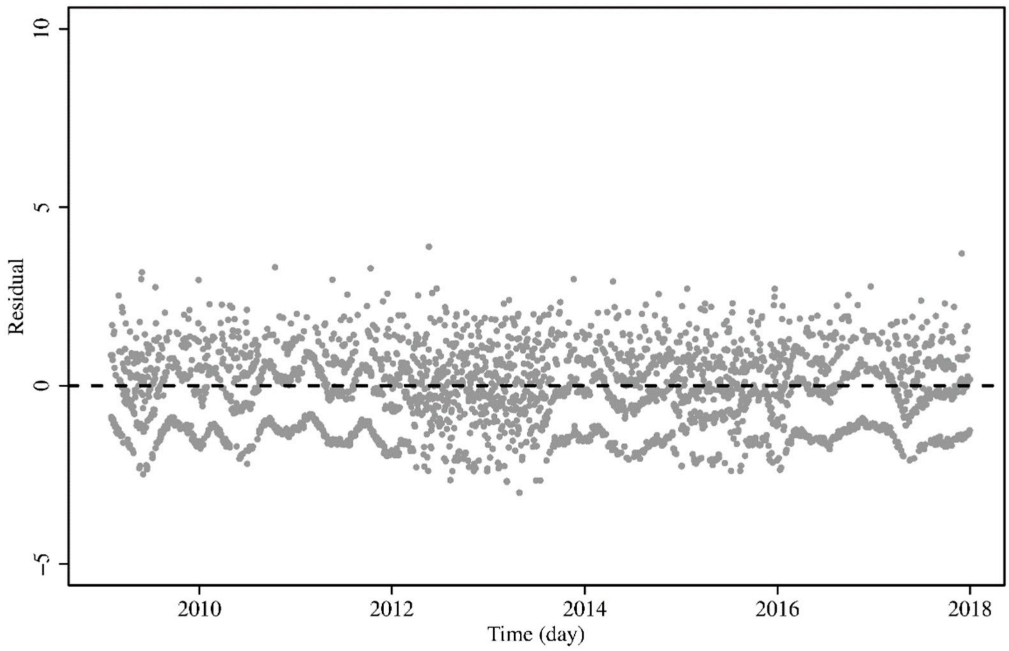

Figure S2 The residual graph of DLNM.
